# Inherited Variants in Regulatory T Cell Genes and Outcome of Ovarian Cancer

**DOI:** 10.1371/journal.pone.0053903

**Published:** 2013-01-30

**Authors:** Ellen L. Goode, Melissa DeRycke, Kimberly R. Kalli, Ann L. Oberg, Julie M. Cunningham, Matthew J. Maurer, Brooke L. Fridley, Sebastian M. Armasu, Daniel J. Serie, Priya Ramar, Krista Goergen, Robert A. Vierkant, David N. Rider, Hugues Sicotte, Chen Wang, Boris Winterhoff, Catherine M. Phelan, Joellen M. Schildkraut, Rachel P. Weber, Ed Iversen, Andrew Berchuck, Rebecca Sutphen, Michael J. Birrer, Shalaka Hampras, Leah Preus, Simon A. Gayther, Susan J. Ramus, Nicolas Wentzensen, Hannah P. Yang, Montserrat Garcia-Closas, Honglin Song, Jonathan Tyrer, Paul P. D. Pharoah, Gottfried Konecny, Thomas A. Sellers, Roberta B. Ness, Lara E. Sucheston, Kunle Odunsi, Lynn C. Hartmann, Kirsten B. Moysich, Keith L. Knutson

**Affiliations:** 1 Department of Health Sciences Research, Mayo Clinic, Rochester, Minnesota, United States of America; 2 Department of Medical Oncology, Mayo Clinic, Rochester, Minnesota, United States of America; 3 Department of Experimental Pathology, Mayo Clinic, Rochester, Minnesota, United States of America; 4 Department of Gynecologic Oncology, Roswell Park Cancer Institute, Buffalo, New York, United States of America; 5 Department of Cancer Prevention and Control, Roswell Park Cancer Institute, Buffalo, New York, United States of America; 6 School of Public Health, University of Texas, Houston, Texas, United States of America; 7 Department of Gynecologic Oncology, Mayo Clinic, Rochester, Minnesota, United States of America; 8 Duke Comprehensive Cancer Center, Duke University, Durham, North Carolina, United States of America; 9 Department of Community and Family Medicine, Duke University Medical Center, Durham, North Carolina, United States of America; 10 Department of Statistical Science, Duke University, Durham, North Carolina, United States of America; 11 University of South Florida College of Medicine, Tampa, Florida, United States of America; 12 Brigham and Women’s Hospital, Boston, Massachusetts, United States of America; 13 University of Southern California, Los Angeles, California, United States of America; 14 National Cancer Institute, Bethesda, Maryland, United States of America; 15 Institute of Cancer Research, Sutton, United Kingdom; 16 University of Cambridge, Cambridge, United Kingdom; 17 Department of Gynecologic Oncology, University of California, Los Angeles, California, United States of America; 18 H. Lee Moffitt Cancer Center & Research Institute, Tampa, Florida, United States of America; 19 Department of Immunology, Mayo Clinic, Rochester, Minnesota, United States of America; Baylor College of Medicine, United States of America

## Abstract

Although ovarian cancer is the most lethal of gynecologic malignancies, wide variation in outcome following conventional therapy continues to exist. The presence of tumor-infiltrating regulatory T cells (Tregs) has a role in outcome of this disease, and a growing body of data supports the existence of inherited prognostic factors. However, the role of inherited variants in genes encoding Treg-related immune molecules has not been fully explored. We analyzed expression quantitative trait loci (eQTL) and sequence-based tagging single nucleotide polymorphisms (tagSNPs) for 54 genes associated with Tregs in 3,662 invasive ovarian cancer cases. With adjustment for known prognostic factors, suggestive results were observed among rarer histological subtypes; poorer survival was associated with minor alleles at SNPs in RGS1 (clear cell, rs10921202, p = 2.7×10^−5^), LRRC32 and TNFRSF18/TNFRSF4 (mucinous, rs3781699, p = 4.5×10^−4^, and rs3753348, p = 9.0×10^−4^, respectively), and CD80 (endometrioid, rs13071247, p = 8.0×10^−4^). Fo0r the latter, correlative data support a CD80 rs13071247 genotype association with CD80 tumor RNA expression (p = 0.006). An additional eQTL SNP in CD80 was associated with shorter survival (rs7804190, p = 8.1×10^−4^) among all cases combined. As the products of these genes are known to affect induction, trafficking, or immunosuppressive function of Tregs, these results suggest the need for follow-up phenotypic studies.

## Introduction

Ovarian cancer is the fifth leading cause of cancer death among women in the United States [1]. Five-year overall survival is approximately 45%, and, even with modern surgical and chemotherapeutic strategies, most cases with advanced disease relapse and succumb to the disease [2], [3]. Rare germline BRCA1 or BRCA2 mutations confer improved survival [4]. Common inherited variants could also influence outcome; genome-wide association studies (GWAS) are underway, but have yet to find survival-associated loci [5]. Consideration of novel biological pathways using in-depth analysis of variation in candidate genes holds promise for the identification of prognostic genetic factors.

Several studies demonstrate the importance of the immune system in ovarian cancer outcome. For example, in one report, cases with evidence of CD3 T cell tumor infiltration (approximately one-half of the cases studied) showed improved progression-free and overall survival [6]. Subsequent studies have refined our understanding of tumor-infiltrating T cells, including one showing that CD8^+^ T lymphocytes are the primary sub-population of T cells associated with better survival [7]. Along with the finding that tumor antigen-specific T cell responses can be detected in cases, these results suggest that anti-tumor immunity is elicited against ovarian cancers and impacts the clinical course of the disease [8]. Despite this generation of an immune response, however, anti-tumor immunity is counterbalanced by an immune suppressive microenvironment [9]. Of immune suppressive mechanisms, CD4^+^ regulatory T cells (Tregs) are a primary means of immune evasion in ovarian cancers; these are CD4^+^ T lineage cells whose primary function is immune regulation [10]. In collaboration with others, we first suggested a role of tumor-infiltrating Tregs in ovarian cancer pathogenesis, reporting higher levels of CD4^+^ Tregs, measured with immunofluorescence, among cases with poorer survival [11]. Subsequent work supports the importance of CD4^+^ Tregs in ovarian cancer pathogenesis and outcome [7], [12]. For example, the presence of CD4^+^ Tregs appears to influence the anti-tumor activity of tumor-infiltrating cytotoxic CD8^+^ T cells [7]. CD4^+^ Tregs block both adaptive and innate immune effectors by cell contact mechanisms as well as by soluble mediators [13], [14]. Soluble mediators of suppression commonly associated with CD4^+^ Tregs include IL-10 and TGF-β, both of which block T cell proliferation and cell-mediated immunity [15], [16], [17]. Other cell surface molecules implicated in suppressing the immune response include B7-H1 (CD274), GITR (TNFRSF18), LAG-3, CTLA-4, and surface-bound TGF-β [18], [19], [20].

Because of the importance of CD4^+^ Tregs and a role for inherited factors in outcome, we assessed whether common inherited variation related to CD4^+^ Treg-related genes was associated with ovarian cancer outcome following standard of care therapy. Specifically, we assessed 54 genes key to the induction, trafficking, or immunosuppressive functions of CD4^+^ Tregs. Utilizing data from a novel candidate gene study combined with existing data, we studied polymorphisms that have been shown to affect the expression of or to tag inherited variation in these genes. Variants associated with outcome in multiple study populations could shed light on immunosuppressive mechanisms in ovarian cancer.

## Materials and Methods

### Ethics Statement

Protocols were approved by the appropriate institutional review boards (Mayo Clinic Institutional Review Board, Roswell Park Cancer Institute Institutional Review Boards, Duke University Institutional Review Boards, National Cancer Institute Institutional Review Boards, Moffitt Cancer Center Institutional Review Boards) or ethics panel (Royal Marsden Hospital ethics panel, University of Cambridge ethics panel, University College London ethics panel). All participants gave written informed consent.

### Candidate Gene SNP Array

Fifty-four genes of key relevance to the biology of Tregs (ACVR2B, AGTR1, CCL11, CCL17, CCL19, CCL2, CCL20, CCL22, CCL3, CCL4, CCL5, CCR4, CCR6, CCR7, CCR8, CD274, CD46, CD80, CD86, CXCL10, CXCL13, CXCR5, DUSP4, EGR2, GPR83, IDO1, IKZF2, IKZF4, IL10RB, IL15RA, IL2RB, IL6ST, IL9, INHBA, INHBB, IRF4, ITGAE, KLF10, LAG3, LRRC32, MDFIC, NRP1, PDCD1, PLAG1, PRNP, RGS1, RGS16, SH3BGRL2, SLC22A2, SMAD3, SOCS2, TNFRSF18, TNFRSF4, and TNFRSF9) were chosen for study (Table S1). The relevance of these genes was established from a PubMed [21] database search which revealed published information that either directly showed or suggested a role for the respective gene products in the induction, immune suppressive function, or trafficking of Tregs. Eighty-five SNPs associated (p<10^−6^) with lymphocyte mRNA expression of one or more candidate genes (eQTL SNPs) were included [22]. In addition, within five kb of each gene, SNPs tagging other SNPs with minor allele frequency (MAF) ≥0.05 at r^2^≥0.9 were identified from 60 European-American participants in the Low-Coverage Pilot of the 1000 Genomes Project (for 53 genes) [23] or the SeattleSNPs Variation Discovery Resource (for CCL2) [24], whichever was most informative. Selected tagSNPs (N = 1,451) were optimized to include SNPs with appropriate design scores for an Illumina Goldengate BeadArray Assay, predicted functionality, and to include more than one tagSNP for large groups of correlated SNPs [25]. Additional SNP information is in Table S2.

### Candidate Gene Study Participants and Genotyping

Cases genotyped on the Treg custom SNP array included women with pathologically-confirmed invasive primary epithelial ovarian, peritoneal, or fallopian tube cancer enrolled at the Mayo Clinic and Roswell Park Cancer Institute (RPCI). Mayo Clinic cases (N = 905) were ascertained between December 1999 and November 2010 into the Mayo Clinic Ovarian Cancer Case-Control Study (MAY) or the Mayo Clinic Case-Only Ovarian Cancer Study (MAC) and included women aged 20 years or above enrolled through Mayo Clinic’s Divisions of Gynecologic Surgery and Medical Oncology. Sixty eight percent of these cases were enrolled within a week of diagnosis (median time from diagnosis to recruitment was zero days). RPCI cases (N = 167) were residents of Western Pennsylvania, Eastern Ohio, or Western New York, aged 25 years or above and ascertained between January 2004 and May 2009 within six months of diagnosis through Roswell Park Cancer Institute’s Divisions of Gynecological Surgery and Oncology. These cases were enrolled with a median time from diagnosis to recruitment of 80 days. At both sites, DNA was extracted from 10 to 15 mL fresh peripheral blood (using Gentra AutoPure LS Purgene salting out methodology at Mayo Clinic and FlexiGene DNA Kit methodology at RPCI), stored at −80°C, and bar-coded to ensure accurate processing. A total of 1,072 participants were genotyped using a custom Illumina Goldengate BeadArray Assay along with 24 duplicates and 24 HapMap CEU replicates (8 trios). Concordance among study duplicates was 99.998%, no SNPs had unresolved replicate or Mendelian errors, and the mean genotype call rate was 99.88%. Samples with genotyping failure (N = 22) or call rate <97% (N = 11) were excluded as well as samples found to be incorrectly plated (N = 1) or from cases deemed ineligible due to non-epithelial disease (N = 5), borderline tumor behavior (N = 34), or enrollment more than one year prior to diagnosis (N = 5). SNPs with genotyping failure (N = 162), call rate <95% (N = 19), or HWE <0.0001 and poor clustering (N = 15) were excluded. Thus, analyses were based on 994 cases and 1,340 SNPs.

### Genome-Wide Association Study

We also analyzed data from the Follow-up of Ovarian Cancer Genetic Association and Interaction Studies (FOCI) collaboration which is part of the National Cancer Institute GAME-ON Post-GWAS initiative and is described elsewhere [26], [27]. In brief, participants were 2,167 self-reported white invasive ovarian cancer cases enrolled in the North Carolina Ovarian Cancer Study (NCO), the NCI Ovarian Case-Control Study in Poland (POL), the Royal Marsden Cancer Study (RMH), the UK Studies of Epidemiology and Risk Factors in Cancer Heredity Ovarian Cancer Study (SEA), the Tampa Bay Ovarian Cancer Study (TBO), and the UK Familial Ovarian Cancer Registry and the UK Ovarian Cancer Population Study (UKR+UKO). Genotyping used Illumina 317 k or 610-Quad Infinium Arrays with imputation to HapMap v 26 using dosage values obtained from the MACH software package [28]. Data on 820 SNPs were available on all cases.

### Statistical Analysis

We used Cox proportional hazards regression accounting for left truncation to estimate hazard ratios (HRs) and 95% confidence intervals (CIs) for association with overall survival. Survival time was defined as time from diagnosis of ovarian cancer until death from any cause or last follow-up. For each SNP, HRs with 95% CIs were estimated per-allele (i.e., 0, 1, or 2 copies of minor allele), analogous to the Armitage test for trend for binary endpoints. Log-additive Cox proportional hazards regression models were adjusted for study site (MAY+MAC, RPCI, POL, NCO, RMH, SEA, TBO, UKO+UKR), age at diagnosis (<50 years, 50–69 years, >70 years), tumor stage (I or II, III or IV, unknown), tumor grade (low, high, unknown), and race (white, non-white, unknown), modeling direct genotype calls for MAY+MAC and RPCI and imputed allele dosage values where appropriate for FOCI participants. Heterogeneity of HRs across study site was formally examined using study-by-SNP interaction terms and performing likelihood ratio tests; no correction for multiple testing was performed.

### Tumor Expression Quantitative Trait Locus (eQTL) Analysis

For 54 genotyped Mayo Clinic cases (33 serous, nine clear cell, eight endometrioid, four mucinous), expression analyses were also performed. Tumor RNA was isolated from fresh frozen samples, using the Qiagen RNEasy protocol and quantitated using a Nanodrop Spectrophotomer (Agilent Technologies, Santa Clara, CA). Total RNA (750 ng) of high quality (RNA integrated number >8.0) was labeled with cyanine 5-CTP or cyanine 3-CTP, using the Low RNA Input Fluorescent Linear Amplification Kit (Agilent Technologies), purified on RNeasy Mini columns (Qiagen), and hybridized to Agilent whole human genome 4×44 K expression arrays (using a mixed reference containing 106 tumor samples). Slides were scanned using the Agilent 2565BA Scanner, and data were normalized using Agilent’s error model and exported by the Agilent Feature Extraction Software (version 7.5.1). Data in the form of the log ratios of signals from individual tumors to signals from the reference mix were used for analysis. For genes with SNPs that were associated with survival at p<0.001, association between genotype and expression probes was assessed using Wilcoxon rank-sum tests.

## Results

The nearly 1,000 invasive ovarian cancer cases genotyped in a Treg custom SNP array and approximately 2,600 cases in the FOCI collaboration demonstrated the expected distributions of mortality, age, and clinical features (Table 1). 1,529 deaths were observed during a median follow-up of 5.4 years. In combined analyses of all cases and case groups defined by histology, eight SNPs yielded p<0.001 including six independent at r^2^<0.95 (Table 2). Results were similar when restricted to 2,518 cases with complete data on stage, grade, and histology. At each SNP, minor alleles were associated with poorer ovarian cancer survival. The most statistically significant association was between survival following clear cell ovarian cancer (N = 217) and minor alleles at an uncommon intronic SNP in the regulator of G-protein signaling 1 (RGS1), suggesting an almost three-fold increased risk of death (p = 2.7×10^−5^).

**Table 1:** Distributions of Ovarian Cancer Clinical Characteristics by Study.

	MAY+MAC(N = 873)	RPCI(N = 121)	NCO(N = 492)	POL(N = 210)	RMH(N = 143)	SEA(N = 1,087)	TBO(N = 212)	UKO+UKR(N = 527)	Total(N = 3,665)
Vital status at last follow up									
Alive	450 (52%)	74 (61%)	264 (54%)	95 (45%)	57 (40%)	719 (66%)	118 (56%)	359 (68%)	2,136 (58%)
Deceased	423 (48%)	47 (39%)	228 (46%)	115 (55%)	86 (60%)	368 (34%)	94 (44%)	168 (32%)	1,529 (42%)
Age at diagnosis									
<50 years	126 (14%)	19 (16%)	119 (24%)	66 (31%)	45 (31%)	260 (24%)	35 (17%)	94 (18%)	764 (21%)
50–69 years	484 (55%)	71 (59%)	299 (61%)	117 (56%)	97 (68%)	780 (72%)	134 (63%)	332 (63%)	2,314 (63%)
70+ years	263 (30%)	31 (26%)	74 (15%)	27 (13%)	1 (1%)	47 (4%)	43 (20%)	101 (19%)	587 (16%)
Histology									
Serous	638 (79%)	89 (82%)	294 (65%)	93 (65%)	55 (48%)	502 (53%)	139 (74%)	282 (60%)	2,092 (65%)
Endometrioid	106 (13%)	11 (10%)	80 (18%)	28 (20%)	26 (23%)	201 (21%)	27 (14%)	91 (19%)	570 (18%)
Clear cell	46 (6%)	1 (1%)	58 (13%)	9 (6%)	17 (15%)	109 (12%)	11 (6%)	46 (10%)	297 (9%)
Mucinous	22 (3%)	7 (6%)	20 (4%)	13 (9%)	16 (14%)	129 (14%)	12 (6%)	53 (11%)	272 (8%)
Other/unknown	61	13	40	67	29	146	23	55	434
Stage									
Stage I/II	176 (20%)	25 (23%)	160 (33%)	56 (39%)	0	512 (61%)	50 (24%)	216 (47%)	1,195 (38%)
Stage III/IV	692 (80%)	84 (77%)	330 (67%)	86 (61%)	0	326 (39%)	155 (76%)	247 (53%)	1,920 (62%)
Unknown	5	12	2	68	143	249	7	64	550
Grade									
Low grade	116 (14%)	37 (33%)	202 (42%)	61 (47%)	43 (54%)	442 (55%)	51 (24%)	168 (43%)	1,120 (37%)
High grade	724 (86%)	76 (67%)	278 (58%)	69 (53%)	36 (46%)	365 (45%)	156 (75%)	225 (57%)	1,929 (63%)
Unknown	33	8	12	80	64	280	5	134	616
Self-reported race									
White	767 (98%)	112 (93%)	492 (100%)	210 (100%)	143 (100%)	1,087 (100%)	212 (100%)	527 (100%)	3,550 (99%)
Non-white	13 (2%)	8 (7%)	0	0	0	0	0	0	21 (1%)
Unknown	93	1	0	0	0	0	0	0	94

**Table 2:** Regulatory T Cell SNPs Associated with Overall Survival (p<0.001).

Case Group	Gene	SNP	MAF	Location	HR (95% CI)	p-value
Clear Cell (N = 217)	*RGS1*	rs10921202	0.07	Intron	2.93 (1.77–4.84)	2.7×10^−5^
Mucinous (N = 272)	*LRRC32*	rs3781699	0.35	3′ UTR	2.32 (1.45–3.71)	4.5×10^−4^
		rs7944357	0.44	Intron	2.04 (1.34–3.10)	8.3×10^−4^
	*TNFRSF4/TNFRSF18*	rs3753348	0.05	Intergenic	3.41 (1.65–7.05)	9.0×10^−4^
Endometrioid (N = 570)	*CD80*	rs13071247	0.14	Intron	1.73 (1.26–2.39)	8.0×10^−4^
All Cases (N = 3,655)	*CD80*	rs7804190	0.37	*MAD1L1*	1.14 (1.06–1.23)	8.1×10^−4^

Adjusted for study site (MAY+MAC, RPCI, POL, UKO+UKR, TBO, NCO, RMH, SEA), age at diagnosis (<50 years, 50–69 years, >70 years), tumor stage (I or II, III or IV, unknown), race (white, non-white, unknown), and tumor grade (low, high, unknown); linkage disequilibrium reduced to r^2^<0.95; MAF, minor allele frequency.

Among 272 mucinous ovarian cancers, minor alleles at four SNPs in leucine rich repeat containing 32 (LRRC32) were associated with greater than two-fold poorer survival. Three of these SNPs (rs3781699, rs3197153, rs3781701) were highly correlated (r^2^≥0.97), thus only one of these is presented in Table 2 (rs3781669 p = 4.5×10^−4^). A fourth SNP, LRRC32 SNP rs7944357, was only modestly correlated with this three-SNP cluster (r^2^≤0.26), yet was also associated with survival among cases with mucinous ovarian cancer (p = 8.3×10^−4^). One other SNP rs3753348 showed association with a more than three-fold risk with survival in mucinous ovarian cancer (Table 2); this SNP resides in the 5 kb region between tumor necrosis factor receptor superfamily members 4 and 18 (TNFRSF4 and TNFRSF18) on chromosome 1.

Among 570 endometrioid ovarian cancer cases, minor alleles at an intronic CD80 tagSNP rs13071247 were associated with a 73% increased risk of mortality (p = 8.0×10^−4^, Table 2). In addition, analysis of all cases regardless of histology suggested that an eQTL SNP for CD80 (rs7804190) was associated with 14% shorter survival time (p = 8.0×10^−4^). The rs7804190 eQTL, which resides intronic to MAD1 mitotic arrest deficient-like 1 (yeast) (MAD1L1), is of interest because genotype has been found to correlate with expression of CD80 (p = 6.0×10^−7^), as well as polymerase (DNA directed), beta (POLB), proline-serine-threonine phosphatase interacting protein 2 (PSTPIP2), and KIAA1128 (p<10^−6^) in lymphoblastoid cell lines [22]. Of note, no SNPs were associated with survival following serous ovarian cancer (p<0.001), the most common and most lethal histologic subtype. Tests for interaction revealed no site-specific heterogeneity of association for any of the SNPs in Table 2 (p>0.05 for each).

**Figure 1:**
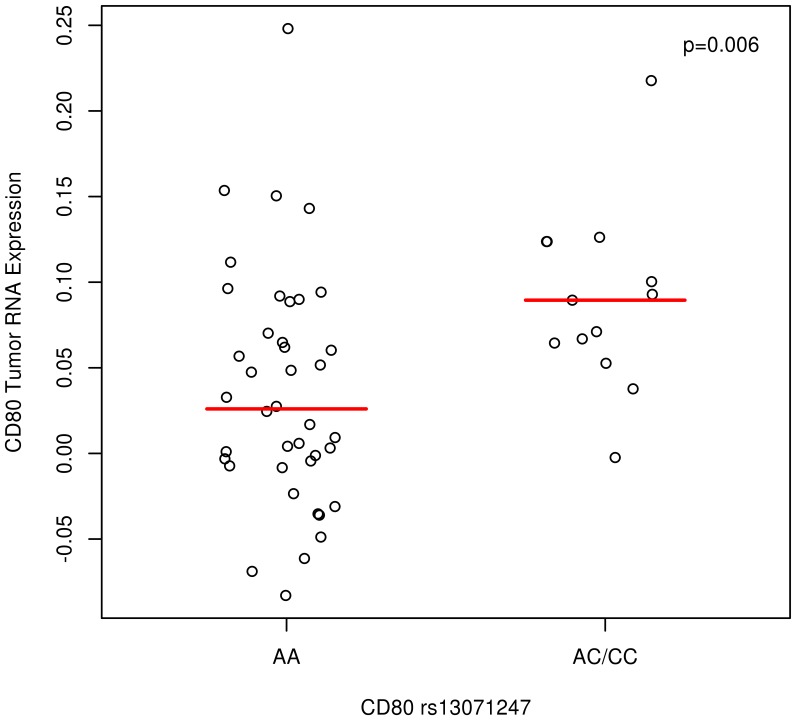
*CD80* rs13071247 Genotype and Tumor Expression. Among N = 54 MAY+MAC invasive ovarian cancer cases, Agilent whole human genome 4×44 K expression array probe A_24_P155632; CD80 log_2_ ratios of tumor versus reference RNA expression (y-axis) versus genotype (x-axis); dashes indicate median; each symbol represents a unique patient; data points are jittered so all data are visible.

To explore potential mechanisms of the observed SNPs associated with ovarian cancer survival, we examined tumor RNA expression data on 54 Mayo Clinic cases and correlated expression with genotype at the most suggestive survival-associated SNPs described above. At CD80 rs13071247, an association was observed such that heterozygotes and minor allele homozygotes (AC and CC genotypes) had slightly increased tumor expression (median fold change  = 1.04 on the raw scale, 0.06 on the log_2_ scale) than cases with AA genotype (p = 0.006; Figure 1). No other associations between genotype and tumor RNA expression were observed at p<0.05.

## Discussion

Previous studies support an important role for Tregs in ovarian cancer which appear to foster an immune suppressive microenvironment. Here, we analyzed ovarian cancer survival in relation to inherited Treg genotypes using a combination of customized genotyping and integration of existing genotypes from a collaborating GWAS. While no SNPs yielded suggestive results among the more common serous cases, poorer survival was associated with minor alleles at SNPs in RGS1 (clear cell), LRRC32 and TNFRSF18/TNFRSF4 (mucinous), and CD80 (endometrioid). For the latter, additional data support a CD80 genotype association with CD80 tumor RNA expression. Combined analysis of multiple independent datasets provides greater statistical power than separate discovery and replication analyses [29]; thus, we used this study design and examined the possibility of heterogeneity of results across studies. As no statistically significant heterogeneity across studies was observed, our inference is based on this combined, most powerful approach. It is worth noting that we examined a relatively large number of SNPs across 54 Treg-associated genes and across different histologic subtypes, and therefore we acknowledge that multiple testing issues may exist; some of our highlighted results could indeed be false positive associations. The fact that the genes examined were chosen based on an a priori role in ovarian cancer and that we concentrated only on SNP associations with p-values less than 0.001 lessens, but does not entirely eliminate, this possibility. Nonetheless, this work highlights particular Treg-related genes of interest.

CD80 has been extensively studied for its role in immune responses, yet no focused analysis of SNPs and ovarian cancer outcome has been reported, to our knowledge. It acts as a ligand for both CD28 and CTLA4, leading to proliferation and anergy in naïve T cells, respectively [30], [31], [32]. CTLA4 is constitutively expressed in Tregs, where it is important in suppressing immune responses through a variety of proposed mechanisms, including activation of the indoleamine-2,3-dioxygenase pathway in dendritic cells (DCs) and inhibition of interactions between activated T cells and DCs [33], [34], [35]. We observed that an intronic tagging CD80 SNP was associated with poorer survival of endometrioid cases and with increased tumor CD80 expression, and, among all cases, we observed that an eQTL SNP on another chromosome which associated with CD80 lymphocyte expression was also associated with poorer survival. Altogether, these data suggest that CD80 expression may be in part driven by inherited factors and may lead to increased immune suppression and poorer outcome.

Of interest to clear cell ovarian cancer, the gene product of RGS1, RGS1 or BL34, is a member of the RGS protein family whose members are involved in regulation of G-protein signaling. Specifically, they are GTPase-activating proteins that limit the duration of G-protein signaling [36], [37]. Lymphocyte migration to chemotactic signals are mediated by G-protein signals and previous studies have found that Tregs do not respond as well to chemokine signaling as naïve T cells [38]). Furthermore, RGS1 is more highly expressed in Tregs and expression is inversely correlated with migration [38]. Interestingly, RGS1 gene expression is increased in activated Tregs, and this is mediated by binding of the Treg transcription factor FOXP3 to the RGS1 gene [39]. Associations of RGS1 SNPs have also been observed in numerous T cell-mediated autoimmune diseases, including type 1 diabetes, celiac disease, and multiple sclerosis [40], [41], [42], [43]. Based on these prior studies, it is tempting to speculate that genetically determined RGS1 levels may regulate Treg infiltration into clear cell ovarian cancers and thus contribute to outcome.

A SNP of possible relevance to mucinous disease is in LRRC32 which encodes GARP, a transmembrane protein expressed specifically on naturally occurring activated Tregs, but not resting Tregs [44], [45]. GARP has been shown to be a receptor for inactive, latency-associated peptide (LAP) bound TGF-β [46], [47]. GARP does not induce activation of latent TGF-β; however, it may function in infectious tolerance, converting FOXP3^−^ cells to suppressive FOXP3^+^ cells [46], [48]. Additionally, GARP is part of a positive feedback loop with FOXP3 in Tregs, which are known to maintain a suppressive tumor microenvironment and prevent an effective immunological response [45]. Our finding of poorer survival among mucinous cases with minor alleles at an LRRC32 SNP suggests that these cases may have increased immune suppression.

We report an association between risk of death due to mucinous ovarian cancer and a SNP in a gene cluster containing TNFRSF18 and TNFRSF4. TNFRSF18 encodes GITR, a co-stimulatory molecule present constitutively on Treg cells and upregulated on naïve T cells after stimulation. Reports are conflicting as to the role of GITR, as it has been reported both to increase [49], [50] and to abolish the suppressive function of Tregs [51], [52]. While the specific mechanism of GITR action remains controversial, it is clear that it modulates Tregs. TNFRSF4 encodes OX40 (CD134), and signaling through OX40 reduces the ability of Tregs to act as suppressor cells by decreasing expression of FOXP3 [53], [54]. Reduced FOXP3 results in decreased miR155 and a subsequent increase in SOCS1 (suppressor of cytokine signaling 1). SOCS1 is a component of a negative feedback loop for IL-2 signaling cascade; increased expression of SOCS1 results in a need for increased IL-2 levels for survival of Tregs [55]. Thus, OX40 signaling in Tregs can modulate suppressor functions both by decreasing their effector function and by requiring higher amounts of IL-2 for continued survival and activation. The intergenic SNP associated with overall survival in cases with mucinous ovarian cancer may act through either a GITR or OX40 mechanism. Dissecting the role of the germline variants may help to identify mechanisms that could explain why the immune system is not able to mount an effective response to ovarian cancers.

In conclusion, our analysis of 3,662 invasive ovarian cancer cases suggests that inherited variants related to Tregs are associated with ovarian cancer outcome in a subtype-specific manner, even after adjustment for known prognostic features. Our findings underscore the importance of subtype-specific analyses in clinical and epidemiological studies of ovarian cancer, given the established disease heterogeneity, with each histologic subtype expressing different patterns of genetic, epidemiologic and clinical characteristics (reviewed by Karst and Drapkin [56]). Future work should include examination of additional study populations, immunological studies, and correlation of inherited variants with other tumor features, such as levels of Treg infiltration.

## Supporting Information

Table S1 Gene information.
(XLS)

Table S2 SNP information.
(XLSX)
